# Towards a New 3Rs Era in the construction of 3D cell culture models simulating tumor microenvironment

**DOI:** 10.3389/fonc.2023.1146477

**Published:** 2023-04-03

**Authors:** Long Zhang, Weiqi Liao, Shimin Chen, Yukun Chen, Pengrui Cheng, Xinjun Lu, Yi Ma

**Affiliations:** ^1^ Organ Transplant Center, The First Affiliated Hospital, Sun Yat-sen University, Guangzhou, China; ^2^ Zhongshan School of Medicine, Sun Yat-sen University, Guangzhou, China; ^3^ Guangdong Provincial Key Laboratory of Organ Donation and Transplant Immunology, The First Affiliated Hospital, Sun Yat-sen University, Guangzhou, China; ^4^ Guangdong Provincial International Cooperation Base of Science and Technology (Organ Transplantation), The First Affiliated Hospital, Sun Yat-sen University, Guangzhou, China; ^5^ Department of Biliary-Pancreatic Surgery, Sun Yat-sen Memorial Hospital, Sun Yat-sen University, Guangzhou, China

**Keywords:** tumor microenvironment, tumor cells, the three dimensional, cell culture, the two dimensional

## Abstract

Three-dimensional cell culture technology (3DCC) sits between two-dimensional cell culture (2DCC) and animal models and is widely used in oncology research. Compared to 2DCC, 3DCC allows cells to grow in a three-dimensional space, better simulating the *in vivo* growth environment of tumors, including hypoxia, nutrient concentration gradients, micro angiogenesis mimicism, and the interaction between tumor cells and the tumor microenvironment matrix. 3DCC has unparalleled advantages when compared to animal models, being more controllable, operable, and convenient. This review summarizes the comparison between 2DCC and 3DCC, as well as recent advances in different methods to obtain 3D models and their respective advantages and disadvantages.

## 1 Introduction

Despite significant advances in human research on tumor staging, diagnosis and treatment, tumors remain one of the leading causes of death (1). Cancer cells can grow and metastasize rapidly, which is largely attributed to the ability of cancer cells to create a tumor microenvironment (TME) for themselves and progressively modulate it from an anti-tumor response to a tumor-friendly one (2).

Therefore, establishing an experimental model system that accurately mimics the complexity of the TME is essential. Traditional *in vitro* two-dimensional cell culture systems (2DCC) (on planar scaffold) and animal models have been widely used for cancer research. However, 2DCC systems do not mimic natural TME due to a lack of cell-cell communication and interactions of cell-cell and cell-matrix (3), while *in vivo* animal models are expensive, ethically problematic, and challenging to set up as they show difficulties in tracking tumor growth and drug screening (4). To address these limitations, the three-dimensional cell culture system (3DCC) is increasingly developed in research and is now crucial for oncology studies due to its ability to accurately maintain TME without any additional manipulation. In this review, we summarize the comparison between 2DCC and 3DCC, as well as recent advances in different methods to obtain 3D models and their advantages and disadvantages.

## 2 Introduction of the tumor microenvironment

**Figure 1:**
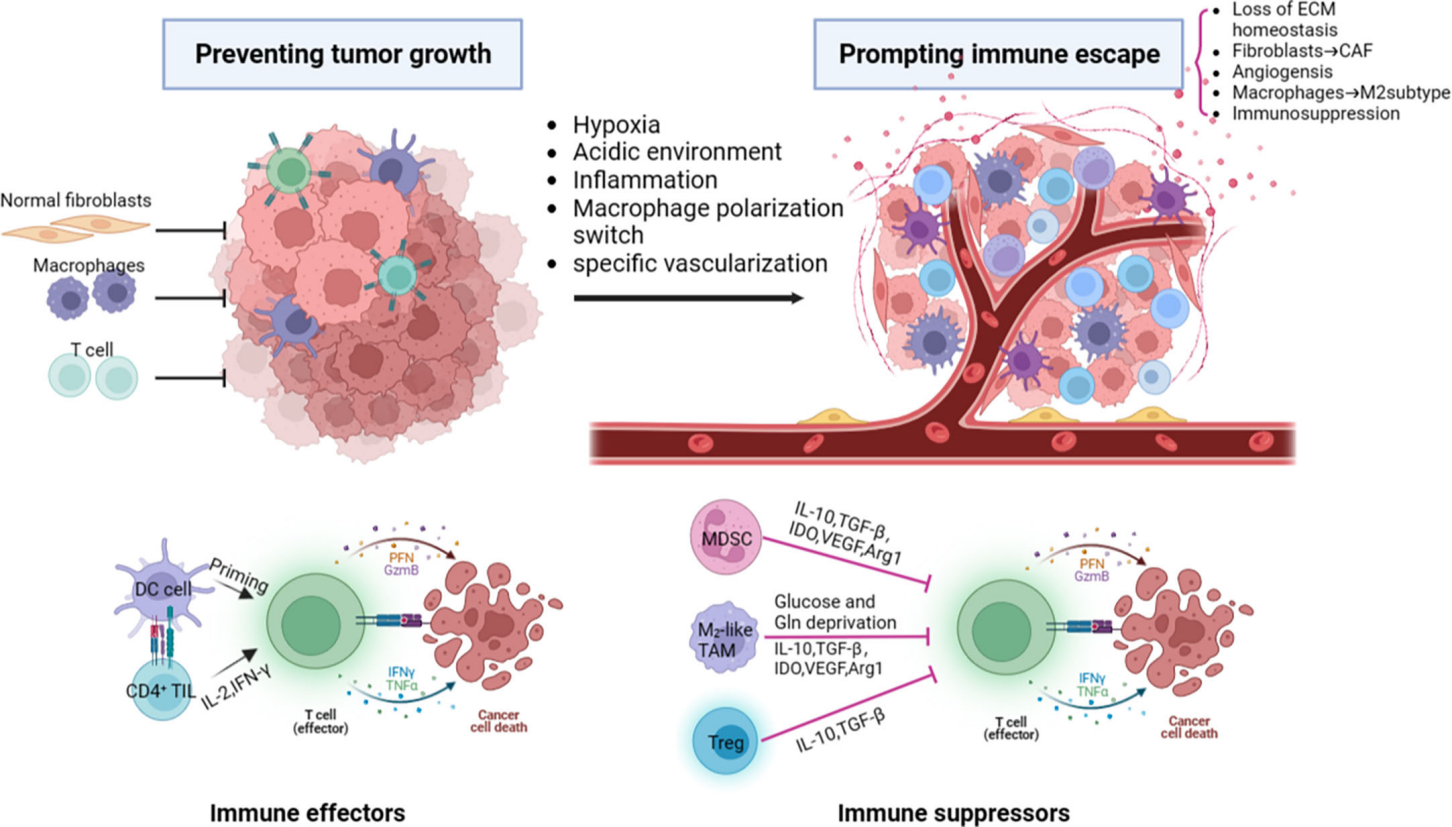
The major components and characteristics related to tumor progression within the tumor microenvironment. ECM, extracellular matrix; CAF, cancer-associated fibroblast; DC, dendritic cells; PFN, perforin; GzmB, granzyme B; IFNγ, interferon γ; TNFα, tumor necrosis factor α; TGF-β, transforming growth factor-β;MDSC, myeloid-derived suppressor cell; IDO, Indoleamine 2,3-dioxygenase; VEGF, vascular endothelial growth factor; Arg1, arginase 1; Gln, glutamine; Treg, regulatory T cell.

TME refers to the cellular environment in which tumor or cancer stem cells reside which has its own unique characteristics compared to the microenvironment of the normal one. These characteristics are important for the tumor immune escape, growth, survival, and metastasis which include hypoxia, acidic environment, inflammatory microenvironment, specific vascularization (**Figure 1**). TME consists of the extracellular matrix (ECM) and various tumor-associated cells such as cancer-associated fibroblasts (CAFs), endothelial cells, adipocytes, and immune cells (5, 6). These cells are located around tumor cells and are energized by the vascular network (7). CAFs can be simply defined as fibroblasts (non-epithelial, non-cancerous, non-endothelial, and non-immune cells) located within or adjacent to a tumor and are the major producer of ECM and various other cytokines in the TME. CAFs have functions of immunosuppression, promoting angiogenesis, producing enzymes that degrade ECM (such as matrix metalloproteinases), and promoting tumor growth and metastasis. However, some CAFs have been shown to inhibit tumor activity (8). Immune cells (T cells, neutrophils, macrophages, etc) play an important role in tumor growth, migration, and immune escape. The pro-tumor inflammation feature within the TME promotes tumor growth by blocking anti-tumor immunity and influent the composition of immune cells within it. Result to the activation of transcription factors in tumor cells, leading to increased inflammation and the production of inflammatory microenvironments adapted to tumor cell growth. Tumor-associated macrophages (TAM) usually divided into M1 type, which mediates antibody-dependent cytotoxic effects (ADCC) to kill tumor cells, and M2 type, which promotes tumor growth, invasion, metastasis and drug resistance. These two cell types can be interconverted (9). Angiogenesis is essential for tumors. Neovascularization provides oxygen and nutrients to the tumor and promotes tumor metastasis. Tumor vascular endothelial cells (TEC) are involved in the metastasis of cancer cells to the neovascular lumen, help generate CAFs, and mediate tumor invasion and metastasis (10). Tumor cells and cancer stem cells (CSCs) secrete molecules that induce a tumor-promoting phenotype, polarizing macrophages to M2 subtype, fibroblasts to CAF, and ECs to TEC (11). ECM is generally defined as the non-cellular component of a tissue that provides metabolic and structural support to its cellular components. Its main components are collagen, proteoglycan, laminin and fibronectin. In the process of tumor progression, a large number of enzymes such as MMP are produced, leading to active remodeling of the extracellular matrix, and changes in collagen degradation or deposition result in loss of ECM homeostasis, which ultimately interferes with cell-cell adhesion, cell polarity and increases growth factor signaling to promote tumor metastasis (12, 13).

## 3 2D vs 3D: Introduction of 3DCC model and its advantages

**Figure 2:**
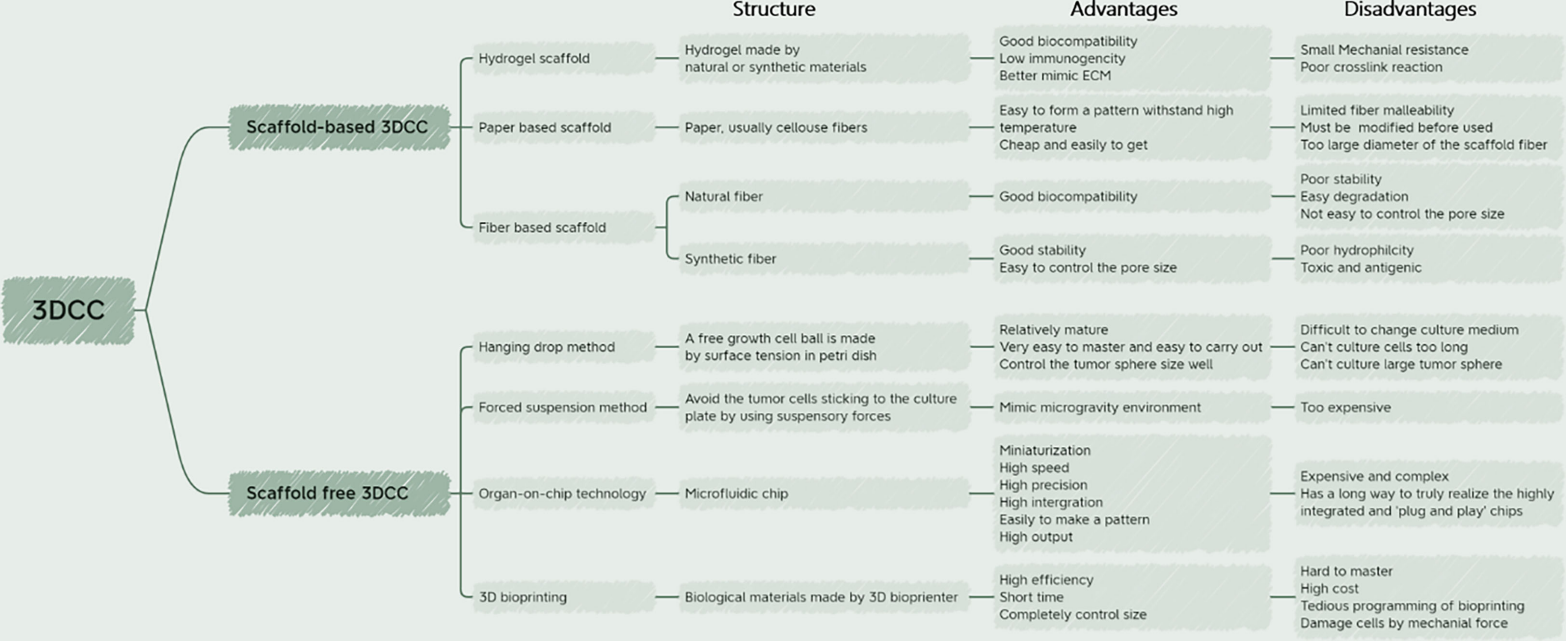
Summary of the 3DCC technique.

Whether the *in vitro* culture model can effectively mimic TME has become an important basis to investigate its practical value. As a traditional *in vitro* cell culture system, 2DCC has long been used in cancer research. However, 2DCC does not mimic the complexity of 3D tissues *in vivo*, nor does it mimic the interaction between tumor cells and TME. Gradients of nutrient and oxygen concentrations are common in TME (14), but cannot be reproduced in 2DCC (15, 16). To address these limitations, 3D cell culture (3DCC) was developed. The 3D tumor sphere model can narrow the gap between 2DCC and *in vivo* tumor model, making the model closer to the real tumor tissue (17) (**Figure 2** and **Table 1**). At present, 3D sphere models can be divided into four types: multicellular tumor sphere (MCTS), neoplastic sphere, tissue-derived tumor sphere (TDTS), and organotypic multicellular sphere (OMS) (20). The cultural methods and biological characteristics of the different types of models are different. MCTS were produced in single-cell suspension cultures in conventional FBS supplemented media without the supply of exogenous ECM. But not all cell lines are capable of producing compact MCTS (20). Tumor spheres were established as amplification models of CSCs in a serum-free medium supplemented with growth factors. It was used to enrich CSCs and cells with stem cell-related characteristics (21). TDTS and OMS were obtained from the tumor tissue department. TDTS were observed in an *in vitro* study of colon cancer cell lines (22). The histological features of OMS are very similar to those of tumors *in vivo*, and capillaries can be maintained for up to 6 weeks (23). MCTS is one of the most commonly used models because it is relatively easy to assemble, possesses reproducibility and ability to mimic tumor cell heterogeneity (24). For the study of tumor initiation, smaller, well-oxygenated spheres (optimal diameter of about 200μm) can be used. In contrast, for studies related to tumor expansion, larger spheres are preferred to mimic the hypoxic and necrotic regions observed in hypovascularized tumors (14). The following is a detailed description of how 3DCC is constructed.

**Table 1:** Comparison of 2DCC and 3DCC (3, 6, 16–19).

	2DCC	3DCC
Morphology	Loss of shape changes and polarization	Actual shape form
Gene expression	To be altered or modified by planar culture or genes	Better representation of tumor gene expression *in vivo*
TME	None	Mimics TME in tumor tissue
Oxygen, nutrients, signaling molecular gradients in TME	None	Controlled by sphere size and molecular osmotic migration rate
Heterogeneity of tumor	Base	Better approximated by various molecular gradients in the TME
Angiogenesis	Only observational	Functional angiogenesis
Cost	Low	High
Multicellular explore	Suitable for immune response studies	Suitable for cell co-culture

## 4 3DCC and tumor initiating cells

Tumor initiating cells play a huge role in tumor malignancy and chemotherapy resistance. The niche of cancer stem cells *in vitro* differs significantly from that *in vivo*. One important aspect of the niche is to maintain stem cells in a quiescent state while simultaneously driving a sufficient number of stem cells into proliferation and differentiation pathways to maintain organs’ function (25). Many signaling pathways that mediate the interaction of normal stem cells with their niche are also involved in the interaction between cancer stem cells and their niches and can promote tumorigenesis and cancer proliferation. Cancer stem cells tend to be quiescent in the body’s milieu interieur, but they exhibit greater proliferative activity *in vitro* than non-cancer stem cells (26).

Currently, the most reliable model for studying cancer stem cells is a 3D assay using an ECM-rich Matrigel, which maintains the growth of heterogeneous layered cancer stem cell cultures. The addition of ECM group stratified adhesins to serum-free medium increases tumor cell growth, self-renewal, and tumorigenic characteristics of glioma cancer stem cells (27). Therefore, the use of 3DCC to study its biological behavior and role in tumors is also a hot topic today. Now some new models are built using 3DCC to study the self-renewing cancer population in depth. For instance, Hubert et al. (28) established CSC cultures derived from the hyperoxia, vegetatively high regions and mixed regions of chronic hypoxia and necrosis regions derived from human glioblastoma. Li et al. (27) constructed a three-dimensional spheroid model of non-small cell lung cancer and used A549 and SK-MES-1 to assess cell growth, migration, drug resistance and other phenomena. In the three-dimensional spheroid model, the commonly used drug tadalafil showed a more pronounced inhibitory effect. Fibrin deposition in the matrix of CRCs proved to be the cause of tumor development. Zhang M et al. (29) used salmon fibrin gel to provide 3D ECM for colon cancer cells and found that 90 Pascal (Pa) fibrin gel was the most effective in isolating and enriching tumor colonies compared to rigid 420 Pa and 1,050 Pa gels. The size and number of colony formations are inversely correlated with gel hardness.

## 5 3D model construction methods for cancer research and its recent progress

In general, 3DCC construction methods can be divided into scaffold-based and scaffold-free models, each with its own set of advantages and disadvantages (30). The following sections will discuss these two methods individually, and provide an overview of the latest advancements in each.

### 5.1 Scaffold-based 3DCC model

3D scaffolds can influence tumor cell-cell and cell-TME interactions by affecting mechanical and biochemical signaling and mimicking the conditions of hypoxia and nutrient deprivation in TME (17). Various forms of materials have been used to construct scaffolds for 3DCC. Depending on the materials, scaffold-based 3DCC can be further classified into hydrogel scaffolds, paper-based scaffolds, and fiber-based scaffolds (15). The support method is simple to operate and easy to disassemble and assemble. However, the disadvantage is that some of the scaffold materials are expensive.

#### 5.1.1 Hydrogel scaffold

Hydrogels consist of one or more different hydrophilic polymers, their unique polymerization mode allows for the free movement of cells and molecules through their pores (31). In the human body, most mammalian cells rely on the extracellular matrix (ECM) for support to carry out life activities. Hydrogels are special because they allow cytokines and growth factors to cross tissue-like gels. Although they contain 95% water, they do provide the solid-liquid level required for cell culture (32). Hydrogels can better replicate the ECM *in vivo*, as they are usually composed of hydrated proteins. They exhibit good biocompatibility and low immunogenicity, but have poor mechanical resistance and crosslinking reactions (17). Hydrogels can be created using natural materials such as collagen, fibrin, hyaluronic acid, and alginate, or synthetic materials like polyethylene glycol (33). The properties of hydrogels, including hydration, porosity, and stiffness, can be fine-tuned by adjusting the components of the material. However, some collagen hydrogels are expensive and have poor renewability (16). Alginate-based hydrogels, like alginate gel beads (ALG beads), are popular 3D substrates for medical applications due to their mild gelation process, biocompatibility, and structural similarity to native tissues (34). Alginate can be cross-linked in the presence of calcium ions and can also be used to degrade scaffolds with sodium citrate to recover cells (35). However, alginate itself does not possess cell adhesion properties due to the lack of interaction with integrins. It also lacks matrix receptors similar to those found in native times, which are important for cell adhesion (34). The pores formed by alginate in the presence of calcium ions are dense and difficult to control accurately, weakening the migration of cells and other biomolecules. Therefore, many improvements have been made to alginate saline gels. Synthetic peptide hydrogels are also a hot research topic. The physicochemical properties of gels can be easily modified by adding or subtracting amino acids or modifying the side chains of amino acid residues (36). There are also many new developments in hydrogel scaffolds of other materials. **Table 2** provides an overview of the latest progress of hydrogel scaffolds.

**Table 2:** New progress of hydrogel scaffolds.

Research purpose	Innovation	Results	Ref.
Model with fibrous matrix and blood vessels	The embedded gel was prepared by combining type I collagen and fibrin	The model can be used to study tumor-matrix interactions in HCC	(37)
Multicellular heterogeneous spheres	Hanging drop method, co-culture of HCC matrix and fibroblasts, encapsulation of collagen gel,	The model is much closer to ECM	(38)
Novel hydrogel scaffolds	HA3P50 scaffold based on hyaluronic acid and poly (methylethylene ether-Alt-maleic acid)	HepG2 cells were protected from the damaging response on 2D medium	(39)
New tumor microsphere model	Biosynthesis of PEG-fibrinogen gels	Similar in size and shape to tumors *in vivo*	(40)
New 3D culture platform	Gelatin and alginate complement each other, PEGDA incorporation controls cross-linking density	A 3D alginate culture platform whose pore size can be changed artificially was made	(35)
Alginate gel microarray	Alginate gel micropores were prepared by electrodeposition of alginate gel on ITO electrodes.	HepG2 spheres were successfully prepared	(41)
Porous alginate beads	Dual aqueous emulsion, controllable pore size, good biocompatibility, can directly encapsulate cells	The activity, proliferation of investigated cells were increased	(42)
Peptide hydrogel	Max8β was used as hydrogel to form nanofibrils through hydrophobic collapse and hydrogen bonding	Custom hydrogels for porosity, permeability and mechanical stability	(43)
Soluble gelatin based cell carrier	Temperature sensitive gelatin microspheres were mixed with alginate saline gel as cell carriers.	A new platform was developed for drug testing and oncology	(44)
New gel sphere	Halo-linked 3D microgels of HA-MA and GelMA in air were prepared on superhydrophobic surfaces.	The shape, size and cell number of the microtumor can be easily controlled.	(45)
New gel matrix	The basement membrane extract was gelatinized with Matrigel to form a new matrix	Better mimic a single tumor in the body	(46)
New alginate hybrid gel beads	Acellular liver matrix and alginate constitute new hybrid gel beads	HCCLM3 cells showed higher cell viability and metastatic potential	(34)
Hydrogel microarray	Application of optical crosslinking technology in micro machining and micro forming.	Produce custom size tumor microspheres	(47)
New Liquid Marble Culture Platform	It forms integrates hydrogel components, replaces liquid with hydrogel and removes hydrophobic shell	The liver specific function and DNA content of HM globules increased after long-term culture	(48)
New hydrogel	Magnetic hydrogels were prepared by combining the assembly of magnetic nanoparticles	Enhances cell-cell interactions and promotes spontaneous formation of multicellular spheres	(49)
New hydrogel	The copolymer reversibly gelatinized in aqueous and redissolved without degrading the synthetic scaffolds	A temperature responsive hydrogel was developed	(50)
3D culture and drug resistance system	The resistance of HepG2 cells to Bio-Pa NPs was detected in 2D and 3D cultures, respectively	HepG2 cells in 3D hydrogels were more resistant to Bio-Pa NPs treatment	(51)
New hydrogel	Low temperature CMCH hydrogel solution gelatinizes rapidly at 37°C	Hydrogels promote cell survival and proliferation, and have good biocompatibility	(52)

#### 5.1.2 Paper base scaffold

Paper is produced by pressing wet cellulose fibers together. Paper-based scaffolds are rigid and can withstand high temperatures, yet they possess some deformability and can be folded into complex geometries, providing pores for cell growth (15, 53). These scaffolds are also hydrophilic with capillary adsorption capacity (54), making them a convenient and cost-effective option for mass production and utilization (53). A convenient 3D culture environment can be created by combining paper-based scaffolds with hydrogel-simulated ECM (55). The gradient of hypoxia and biomolecules in TME can be imitated by stacking paper-based scaffolds. Disassembling the paper-based scaffold facilitates cell harvesting and analysis of the structure and function of cells in the paper-based scaffold without histological sections. The paper platform is used to culture primary cells, tumor cells, patient biopsies, stem cells, fibroblasts, osteoblasts, immune cells, bacteria, fungi, and plant cells. These platforms are compatible with standard analytical assays commonly used to monitor cell behavior. Due to its thickness and porosity, there is no mass transfer limitation to and from cells in the paper scaffold (56). However, paper-based scaffolds have some limitations, such as limited fiber malleability and the need for physical and chemical modification before use in cell culture (54). Furthermore, the diameter of the scaffold fiber is much larger than that of body fibrils (about 500nm), with a minimum diameter of 1mm (15).

#### 5.1.3 Fiber base scaffold

Man-made fiber structures date back thousands of years, and they are used as clothing and decoration in the form of textiles (57). Fiber products are also widely used in filtration, cell culture, composite materials and other processes. Fiber-based scaffolds can be constructed using either natural fibers such as collagen, chitosan, and hyaluronic acid, or synthetic fibers such as polylactic acid, polyglycolic acid, and other degradable polyester polymers. Under the premise of ensuring the porosity of hydrogel and the normal growth of cultured cells, the use of fiber materials to build a platform can act as a scaffold to compensate for the lack of structural rigidity of hydrogels. Adding carbon nanotubes to a hydrogel is a good try (58). Natural fiber scaffolds are known for their good biocompatibility and ability to interact with cell-ECM receptors, which facilitates cell growth. However, these scaffolds have poor stability, are easily degradable, and have a limited ability to control the size of the fiber pore (31). Some fiber production processes, such as electrospinning, use solvents that denature natural fibers (59). In contrast, synthetic fiber scaffolds are stable over a wide range of temperatures and in solution (31). Synthetic fibers are easier to control the pore size and can also be used to mimic the porous structure of ECM (60). However, these fibers may be less hydrophilic, and some may be toxic and antigenic, which can damage cells. Due to these limitations, efforts are being made to enhance their properties while preserving their respective benefits.

In terms of natural materials, Mahmoudzadeh et al. (61) developed collagen-chitosan nanoscaffolds and utilized them to culture 4T1 tumor cells, allowing for the construction of a 3D microenvironment as the tumor cells infiltrated the scaffolds. Koh et al. (62) presented a comprehensive protocol for studying live cell microscopy and immunohistochemistry to quantitatively assess physiological cell-cell contact dynamics. Decellularized natural tissues have also emerged as a source of fibrous scaffolds for cancer research (63), such as decellularized lung scaffolds, which retain the ECM arrangement of the original tissue and allow for better simulation of cell-ECM interactions (64). Tissue engineering can also be employed to construct tumors *in vitro*, as demonstrated by Lu et al. (65), who utilized Tris-trypsin-Triton to treat tumor tissues in multiple steps, creating 3D scaffolds with the ideal spatial arrangement, biomechanical properties, and biocompatibility - a promising approach for modeling the TME.

In terms of synthetic materials, Girard (66) et al. developed the “3P” scaffold, which is produced by electrospinning the block copolymers of poly (lactate-coglycolic acid) (PLGA), polylactic acid (PLA) and mono-methoxy polyethylene glycol (mPEG). Fischbach (67) et al. used polylactide to fabricate fiber scaffolds. Both types of scaffolds are non-toxic to tumor cells and can be produced on a large scale. Additionally, tumor cells grown on these scaffolds exhibit invasion and metastasis characteristics that better replicate the *in vivo* tumor microenvironment. Mazzini (68) et al. and Murakami (69) et al. have both developed 3D tissue culture systems using silicon as a raw material. Mazzini’s team utilized silicon microprocessing technology to produce 3D microarrays for the study of tumor cell invasion. Meanwhile, Murakami’s “Cellbed” culture system is composed of a fibrous polymer made of ultra-fine silica fibers, mimicking the loose connective tissue structure of living organisms. Cancer cells can easily migrate and form 3D structures in this system.

All of the above-mentioned scaffolds are based on natural or artificial materials and are further developed to have more applications as fiber scaffolds.

### 5.2 Scaffold-free 3DCC model

3DCC without scaffolds mainly uses various methods to prevent cell adherent growth and aggregate tumor pellets in culture medium (24). These methods include magnetic force, agitation and rotation, hanging drops, low-adhesion culture plates, and advanced technologies such as microfluidic chips and 3D printing. While scaffold-free 3DCC is suitable for only a limited number of cell types and is initially expensive, it allows spontaneously aggregated cells to form their own ECM (24). Scaffold-free 3DCC does not involve vasculogenesis and can restore the heterogeneity of tumors *in vivo*, thus more closely resembling solid tumors *in vivo*. It is not affected by the shear force of scaffold assembly, nor is it limited by the pore size of scaffold fibers, and can produce controllable size tumor microspheres (70). T Below, we introduce the main methods and recent advances in scaffold-free 3DCC formation.

#### 5.2.1 Hanging drop method

The hanging drop method is a relatively simple technique. Initially, cells are cultured in two dimensions and allowed to adhere to the wall before being digested into monolayer cells. The resulting digested cell culture liquid is then dropped onto the lid of a Petri dish, which is subsequently inverted. The liquid was drooped to form hanging drops through the action of surface tension, and the desired tumor pellets could be formed in the hanging drops. It usually forms in spheres or sheets within 24 hours but may take longer. The length of time required depends on the type of cells (71). This method is well-established, requiring no specialized equipment and is easy to master. The resulting tumor spheres are easy to control in terms of size, with only one sphere formed per drop. Mesenchymal stem cells cultured by the hanging drop system can secrete a large number of potent anti-inflammatory and antitumor factors (72). However, it is difficult to change the culture medium of the traditional hanging drop method, and the culture time of the cells should not be too long. Large tumor spheres cannot be cultivated due to nutrient supply effects (73). Ratnayaka (74) et al. invented a PDMS platform combining the hanging drop method and polydimethylsiloxane (PDMS) scaffold. By using this method, HepG2 cells could be grown to the level of millimeter, which was much higher than the volume of tumor microspheres obtained by the ordinary hanging drop method. Although the pendant method does not mimic tumor angiogenesis and makes it difficult to grow tumors to the size of advanced tumors *in vivo*, it is still possible to obtain larger tumor microspheres with this method, which provides convenience for tumor research.

#### 5.2.2 Forced suspension method

**Figure 3:**
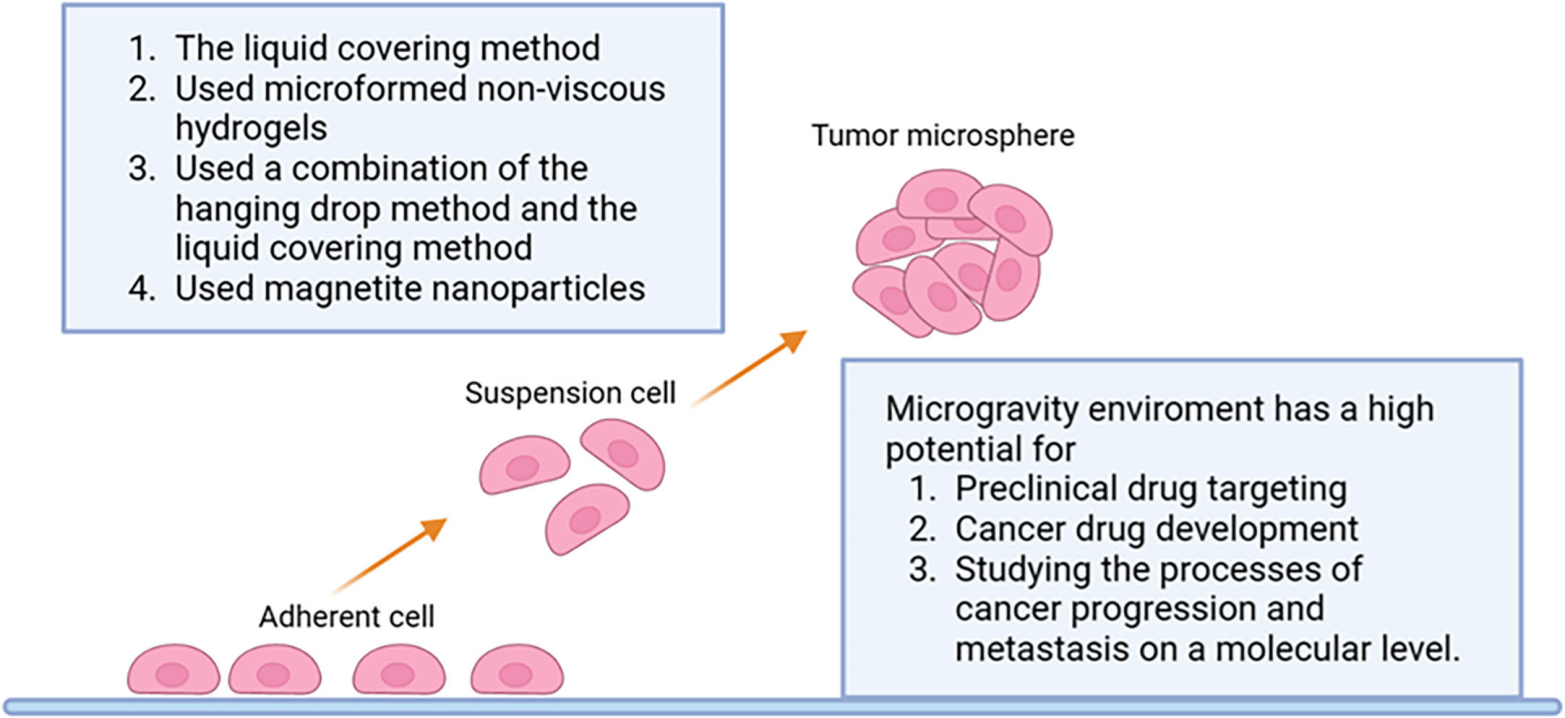
Schematic representation of the forced suspension method.

The forced suspension method is to prevent tumor cells from sticking to the culture plate and forming tumor microspheres by forced suspension. The commonly used method is the liquid covering method, which precoats the surface of the culture plate with low adhesion material in advance to form an ultra-low adhesion culture plate. Tumor cells cannot adhere to the culture plate, so they spontaneously suspend to form tumor microspheres (70). The most commonly used ultra-low adhesion culture plate is a 96-well polystyrene culture plate (70). This method is simple and convenient, and most tumor cells can form tumor microspheres by this method. However, this method cannot control the size and homogeneity of the tumor microspheres formed. Napolitano et al. (75) used microformed non-viscous hydrogels to conduct forced suspension cell culture, and cells spontaneously self-assembled and reached A structural balance controlled by cell-cell interactions. Shao (76) et al. constructed a novel tumor microsphere model by co-culturing melanoma cells and cancer-associated fibroblasts (CSF). In this model, tumor ECM is completely controlled by CSF, which facilitates the study of the interaction between tumor cells and TME. Beheshti (77) et al. used a combination of the hanging drop method and the liquid covering method to form 3D multicellular spheres to test the anticancer effect of Ipomoea purpurea. In addition to these new materials and methods, physical means such as magnetic force and rotation can also be used to achieve the purpose of forced suspension. Magnetic cell suspension is an emerging spheroid-forming technique. To generate spheroids, cells are preloaded with magnetic nanoparticles and then float towards the air/liquid interface within the low-adhesion plate using an externally applied magnetic field to promote cell-cell aggregation and spheroid formation. Glauco R Souza et al. reported a magnetic levitation cell culture model. By controlling the magnetic field, the geometry of the cell can be changed (78). Okochi (79) et al. used magnetite nanoparticles to make cells suspended and gathered in the center of the culture pore through the effect of magnetic force, thus realizing the suspension culture of cells. The rotating cell culture system simulates the microgravity environment by producing laminar flow (80), minimizing the mechanical stress of cell aggregation, making cells grow in suspension and preventing them from sticking to the wall and forming cell spheres. Human mesenchymal stem cells cultured in simulated microgravity have osteogenesis and enhanced adipoiesis (81). This method has been recommended by NASA as an effective tool for modeling microgravity (82). Numerous studies have illustrated the impact of short- and long-term exposure to real and simulated µg on various processes, including differentiation, growth behavior, migration, proliferation, survival, apoptosis, and adhesion, all of which are pertinent to cancer research (83). Thus, the µg-environment facilitates the creation of *in vitro* 3D tumor models, such as multicellular spheroids and organoids, that offer significant potential for preclinical drug targeting, cancer drug development, and the study of cancer progression and metastasis on a molecular level. In conclusion, as depicted in **Figure 3**, the forced suspension method is a widely utilized and well-established technique.

#### 5.2.3 Organ on chip technologies

**Figure 4:**
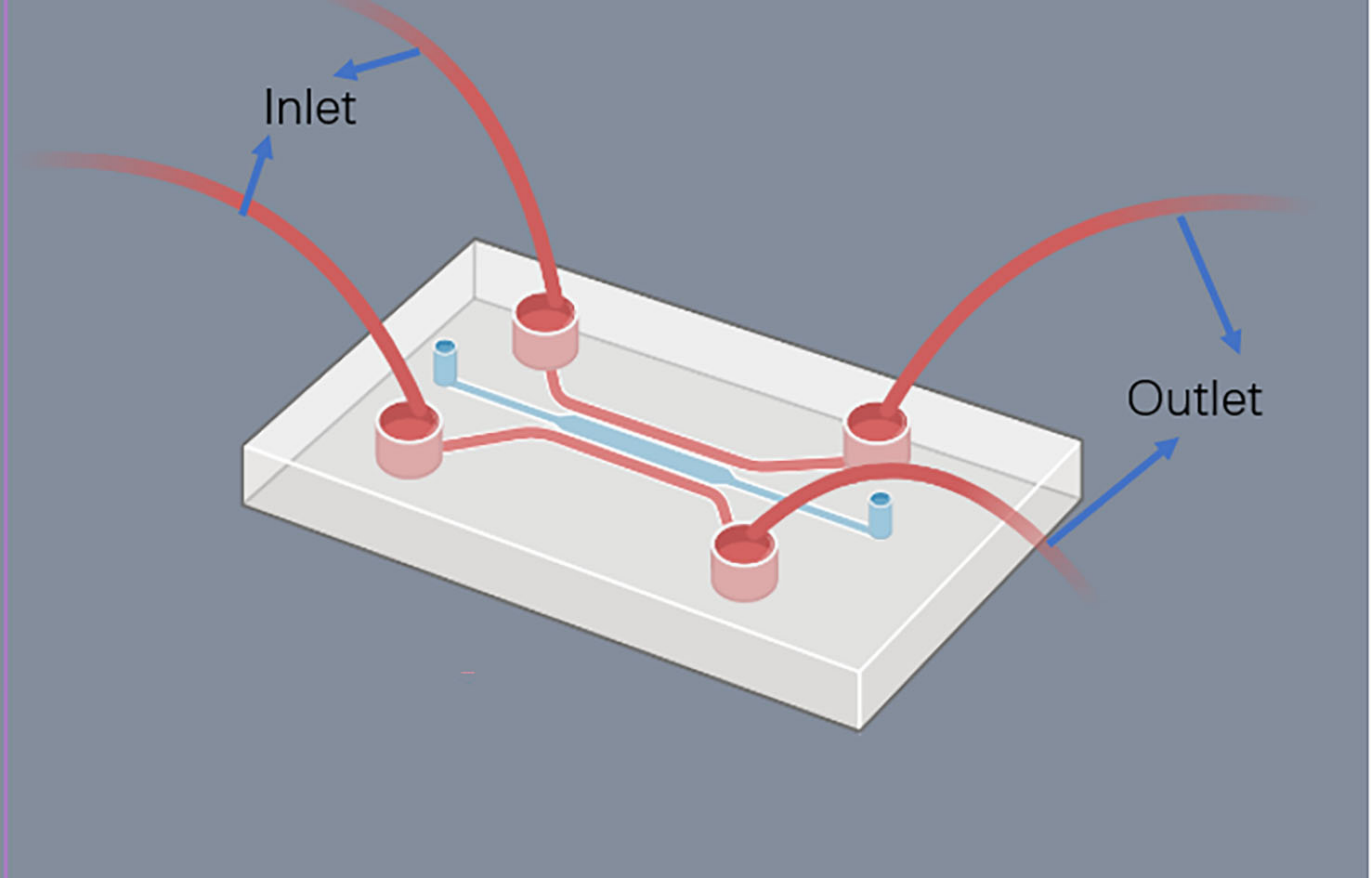
Structure of the microfluid chip.

FDA has recently agreed to assess organ on a chip technology, which has the potential to replace animal models altogether. Cancer on-chip technology typically offers the following solutions (84): (1) 2D chips. Single or multi-chamber chips with controlled substance concentration gradients to study the impact of concentration gradients on cancer metastasis; (2) Lumen chips. Lumen consisting of a patterned 3D matrix, suitable for vascular studies of tumors; (3) Partition chip. Chips are divided into several cells with a separator, capable of culturing different types of cells, making them versatile; (4) Y-type chip. Similar to partition chips but with parallel-matrix compartments operating in co-flow mode; (5) Membrane chip. Capable of creating numerous microchannels with porous membranes, facilitating solute gradients at channel interfaces, cell culture, and transfer observation. Summary of the major organ on chip technologies is now illustrated in **Table 3**. The use of microfluidic chips is an essential component of this technology (**Figure 4**). Being selected as one of the World Economic Forum’s Top 10 Emerging Technologies, Microfluidics integrates sample preparation, reaction, separation, detection, and basic operational units such as cell culture, sorting, and cell lysis (97).

**Table 3:** Summary of the major organ on chip technologies.

Organs	Research type	Results and featured advantages	Ref.
Heart on a chip	Bioprinting 3D microfibrous scaffolds for engineering endothelialized myocardium	1. Combines 3D printing technology and chip technology 2. Precise control of macroisotropic structure of microfibers 3. Improved alignment capable of spontaneous and synchronous contraction.	(85)
	Heart on chip for high throughput drug studies	1. High throughput, reproducible; submillimeter-sized soft elastomer film cantilever arms. 2. Better recapitulation of engineering tissues of *in vivo* physiology and quantification of multiple relevant bioanalyses.	(86)
	Heart on a chip for the neuro–cardiac junction	1. A microfluidic device consisting of two separate cell culture chambers was used to co-culture human neurons and human cardiomyocytes. 3. Summarizes the structural and functional properties of the neuro-cardiac connection. 2. Confirmed the presence of a special structure between the two cell types allowing neuromodulation.	(87)
Lung on a chip	A bovine lung-on-chip	1. Successfully generate a co-culture model of the proximal airway of bovines. 2. May replace *in vivo* experiments. 3. Simulate the blood flow required for systemic administration.	(88)
	A murine lung-on-chip infection model	1. Higher spatiotemporal resolution than animal models by time-lapse imaging technology. 2. The kinetics of host-Mycobacterium tuberculosis interactions at the gas-liquid interface are revealed. 3. The direct role of pulmonary surfactant in early infection is explored.	(89)
	3D Lung-on-Chip Model Based on Biomimetically Microcurved Culture Membranes	1. Reconstruct the main spherical geometry of the cell’s native microenvironment. 2. An innovative combination of three-dimensional microfilm molding and ion tracking technology; 3. May lay the groundwork for other microanatomically-inspired membrane-based OoCs in the future.	(90)
Kidney on a chip	Culture and analysis of kidney tubuloids and perfused tubuloid cells-on-a-chip	1. Establish tubule-like cultures, simulate multi-organ interactions and reduce variability. 2. Control The microtubule microenvironment, increase model complexity. 3. Induces flow-induced shear stress.	(91)
	A kidney organoid-vasculature interaction model using a novel organ-on-chip system	1. Supports culture of renal organoids, which exhibit nephron structure. 2. Organoids cultured on a chip show increased maturity in endothelial populations. 3. Establish the first vascularized renal organoids using microfluidic organ-on-chip under HUVEC co-culture conditions.	(92)
	A kidney on a chip model for drug studies	1. Significant upregulation of organic cationic and organic anion transporters improved drug uptake. 2. Perfused 3D proximal tubule model. 3. OPTEC tubules exhibit higher normalized lactate dehydrogenase release when exposed to known nephrotoxins, which are attenuated with the addition of OCT2 and OAT1/3 transport inhibitors.	(93)
Multi organs on a chip(MOC)	A heart/liver/lung-on-a- chip	1. A highly functioning, perfusion-driven, microfluidic multi-tissue organ-on-a-chip system consisting of liver, heart, and lung organoids. 2. Three bioengineered tissue organoids are able to respond independently or synergistically to various external stimuli. 3. When organoids are combined into a single platform, more complex synthetic responses are observed.	(94)
	A lung/liver-on-a-chip	1. Ligate normal human bronchial epithelial cells cultured at the gas-liquid interface and HepaRG™ liver spheres in a single circuit. 2. MOC allows crosstalk between different organs to be studied to assess the safety and efficacy of compounds better than single cultures. 3. Provide new opportunities to study the toxicity of inhaled aerosols or to demonstrate the safety and efficacy of new drug candidates targeting human lungs.	(95)
	A multi-organ chip with matured tissue niches linked by vascular flow	1. Mature niches of heart, liver, bone and skin tissue are connected by a circulating vascular stream. 2. Summarizes the pharmacokinetic and pharmacodynamic profile of human doxorubicin. 3. Allowing the identification of early miRNA biomarkers of cardiotoxicity.	(96)

Microfluidics is the science of precisely manipulating fluids and particles in sizes ranging from microns to submicrons (98, 99). The use of Polydimethylsiloxane (PDMS) as a basic material for microfluidic chips is popular due to its low cost, good biocompatibility, high oxygen permeability, light transmittance, and convenience (99, 100). Glass and silicon, which are comparatively expensive and difficult to work with, have been gradually replaced by PDMS. Moreover, hydrogels and paper can also be employed to produce microfluidic chips (101). By integrating cell culture, cell separation, cell detection, and other procedures into a small chip, microfluidic technology offers the benefits of miniaturization, high precision, and high integration. Compared with traditional laboratory techniques in the past, the microfluidic platform has the advantages of requiring fewer samples, high sensitivity, rapid control and so on (102).

The microfluidic chip is the main platform of microfluidic technology. It takes a micropipe network as its main structural feature and microfluidics technology to control the flow of liquid in the micropipe as its working principle. Microtubules are filled with living cells, and in this way organs or tissues *in vitro* are constructed to study their physiology and pathophysiology mechanisms (103). Due to the structure of the micron scale, the fluid exhibits and produces special properties in it that are different from those of the macroscopic scale. Hence the development of unique analysis of the resulting performance. Using a microfluidic chip to culture tumor cells can rapidly produce tumor microspheres of controllable size and can also be used for high-throughput analysis of tumor cells at any time. It can be used to simulate the tumor microenvironment, study the invasion and metastasis of cancer cells, simulate tumor angiogenesis, and conduct high-throughput tumor detection (99, 100). Microfluidic chips have the potential to surpass tumor xenograft modeling, which can lead to a significant reduction in animal experimentation and make tumor modeling and research more efficient (104). Despite this, the design and production of microfluidic chips are rather intricate, and there is still a long road ahead before achieving fully integrated and “plug and play” microfluidic chips without costly external auxiliary equipment (105). **Table 4** provides an overview of some of the latest microfluidic chips developed.

**Table 4:** New progress of microfluidic chips.

Research purpose	Innovation	Results	Ref.
Cancer cell migration model	3D collagen barrier is formed through polyelectrolyte composite solidification process	Form 3D aggregates of cells similar to cancer tumors, mimicking the migration of cancer cells *in vivo*	(106)
Microarray cell culture system	1156 square microcontainers, large capacity, to ensure nutrient supply external bioreactor	Preserve the function of rat primary hepatocytes for more than 2 weeks	(107)
New microfluidic platform	The microfluidic platform can realize the microfluidic control with many repetitions and long duration	High-throughput production of tumors of uniform size; Various 3D tumor microspheres produced in the device	(108)
New cell culture methods	Gelatin-based 384-well ready-to-use microscaffold array	It is suitable for a variety of tumor cells and can be used for drug resistance detection and tumor cell culture	(109)
New manufacturing of cell sphere	Construct a multicellular culture platform using acoustic fluid	This produces more than 6000 tumor spheres per operation and shortens the tumor sphere formation time to one day.	(110)
New microfluidic chip	Polydimethylsiloxane is made of a double casting technique with a thermal aging step	The tumor microsphere culture time of up to 4 weeks can observe the reaction of tumor microsphere for a long time	(111)
Novel TME model	Cancer cells in the microcapsule were encapsulated with a hydrogel shell to form a 3D vascularized tumor	The angiogenesis in the 3D microenvironment of human tumor was simulated	(112)
New 3D co-culture model	Panc-1 tumor spheres were co-cultured with pancreatic stellate cells using microarray chips and	The role of pancreatic stellate cells in tumor development and metastasis was studied	(113)

#### 5.2.4 3D bioprinting

**Figure 5:**
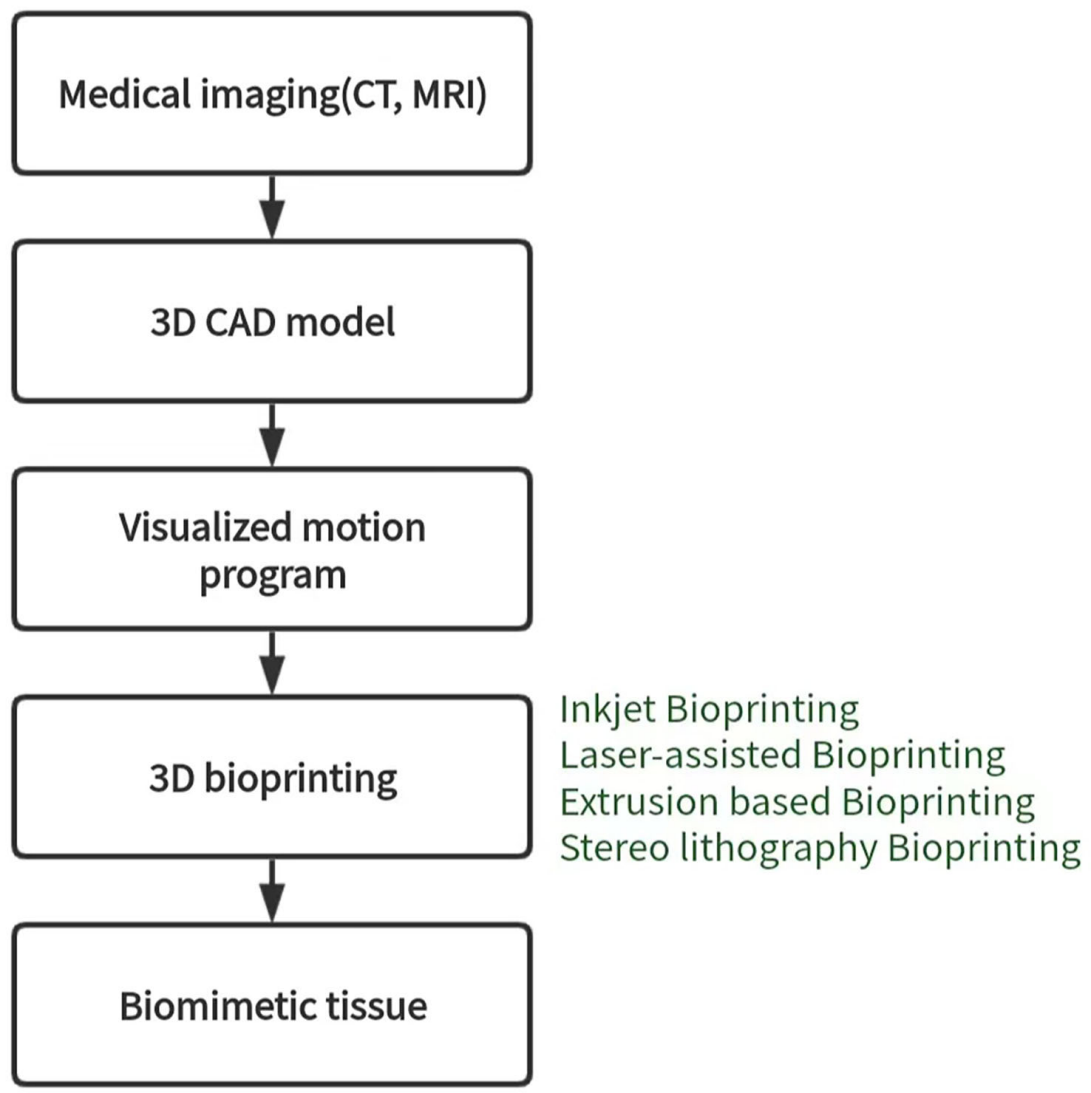
Schematic of 3D bioprinting workflow and different 3D bioprinting techniques.

3D bioprinting is a manufacturing technology that accurately distributes biological materials containing cells to construct three-dimensional living tissues and human organs using a 3D printer. Currently, there are four types of 3D bioprinting technology, including inkjet, laser-assisted, extrusion, and stereo lithography, each with its advantages and disadvantages (114) (**Figure 5**). To construct 3D tumor models *in vitro*, tumors or tumor cells can be combined with TME as printing materials, and bioinks are essential for building effective 3D tumor models. Bioinks are typically biocompatible hydrogels and living cells of interest and play an important role in providing printability of samples (115, 116). Alginate and gelatin are the most commonly used substrates for bioprinting due to their good biocompatibility and mechanical properties. Bioprinting is a new research field. Compared with other cell culture technologies, the biggest advantage of the bioprinting method is that it can form tumor microsphere model with controllable size and shape in a short time, which can be used for various tumor research. However, this method is difficult to master because of its complicated technology, high cost and tedious programming of bioprinting. Inkjet 3D printing is limited due to clogged nozzles, which limits the steady flow of ink, as well as reduced cell viability (72). The mechanical pressure of extrusion printers can also damage cultured cells. Jiang (117) et al. combined alginate and gelatin to form a composite hydrogel similar to a natural tumor matrix. Chen (118) et al. cocultured primary HepG2 human hepatocytes and hepatic stellate cells (HSC) to form spherules. Then the spheres were bioprinted into liver tissue constructs using a Regenova bioprinter to construct a new liver cancer model. Bhattacharjee (119) et al. used packaged granular microgels to make liquid-like solid materials. The material is locally and temporarily fluidized under concentrated applied stress and spontaneously solidifies after the applied stress is removed, facilitating the transport of biomolecules and 3D printing of multicellular structures. In summary, 3D bioprinted cancer models can be valuable as invasion models and serve as an excellent tool to study cancer progression and visualize EMT and metastasis in real-time. Nonetheless, there are still several challenges and limitations that need to be addressed. These include the development of a perfusable, vascularized 3D bioprinted construct, achieving large organ reconstruction *in vitro*, long-term *in vitro* culture, and other related issues.

## 6 Conclusion

3DCC is increasingly important in oncology research as it better mimics solid tumor conditions *in vivo* with minimal use of animal models. Although 3DCC has obvious advantages over 2DCC, 3DCC cannot completely replace 2DCC (120) at present because the monolayer culture equipment is easy to manufacture, the production cost is low and the technology is easy. As summarized in **Figure 2**, 3DCC models have unparalleled advantages in simulating the tumor microenvironment. Although tumor-like 3D cultures allow the expansion of the tumor epithelium, they often lack non-epithelial stromal cells from tumors of origin, limiting their usefulness in addressing therapeutic strategies targeting this compartment (121). With the maturation and development of 3DCC technology, its application in the study of tumor metastasis mechanism, tumor microenvironment, tumor cell-ECM interaction and anti-cancer drug screening will be more extensive and in-depth, making it a promising technology for the future.

## Author contributions

LZ, XL and YM contributed to the manuscript’s composition, literature review, and drafting and finalization of the manuscript. SC, WL and YC contributed to the literature review and search. PC contributed to the manuscript’s drafting and critical review. XL and YM contributed to the approval of the final version of the manuscript.
